# Post-POEM Esophageal Leak Successfully Managed With Endoscopic Vacuum Therapy

**DOI:** 10.14309/crj.0000000000002067

**Published:** 2026-04-06

**Authors:** Mohamed Elshennawy, Sultan Mahmood

**Affiliations:** 1 Gastroenterology and Hepatology Department, Theodor Bilharz Research Institute, Cairo, Egypt; 2 Gastroenterology and Hepatology Department, University of Pittsburgh Medical Center, Pittsburgh, PA

**Keywords:** peroral endoscopic myotomy (POEM), esophageal leak, esophageal perforation, endoscopic suturing, endoluminal vacuum therapy (EndoVAC), endoscopic vacuum therapy (EVT)

## Abstract

Esophageal leak is a well-described complication of endoscopic peroral endoscopic myotomy (POEM). Although various endoscopic techniques exist for managing mucosal defects, their efficacy is not universal, and successful closure is not guaranteed. The endoscopic vacuum therapy (EVT, EndoVAC) has been implemented in the management of mucosal defects. We hereby present a 46-year-old man who developed a persistent esophageal leak after POEM, which was successfully managed with the EndoVAC following multiple unsuccessful closure attempts using conventional methods.

## INTRODUCTION

Peroral endoscopic myotomy (POEM) proved to be an effective and minimally invasive therapy for achalasia since it was first introduced in 2008.^1,2^ Although the procedure is generally safe, complications such as esophageal leaks, although rare, have been described previously. Various endoscopic and surgical approaches have been described for management, including clipping, suturing, and stent placement.^3^ The endoluminal vacuum therapy (EndoVAC) for esophageal perforations has been reported to be effective and successful.^4^ We hereby report a case of post-POEM esophageal leak successfully managed using endoscopic vacuum therapy (EVT).

## CASE REPORT

**Figure 1.**
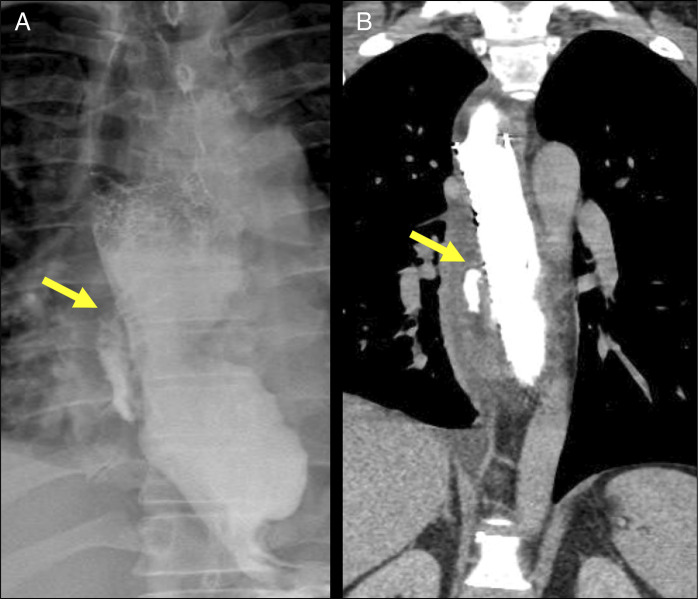
(A) Esophagogram showing a contained leak (arrow). (B) Chest CT with contrast showing a persistent contained leak (arrow).

A 46-year-old man with type II achalasia confirmed by esophageal manometry and barium esophagram. Treatment options, including pneumatic dilation, laparoscopic Heller myotomy with fundoplication, and POEM, were discussed with the patient. The patient opted for POEM, which was performed successfully without any intraprocedural complications. The following day (first day postoperative), the patient developed nausea, bouts of retching, followed by chest discomfort, but no hematemesis or other red flag symptoms. An esophagram was performed and revealed mediastinal contrast extravasation, confirming a contained esophageal leak (Figure 1).

**Figure 2.**
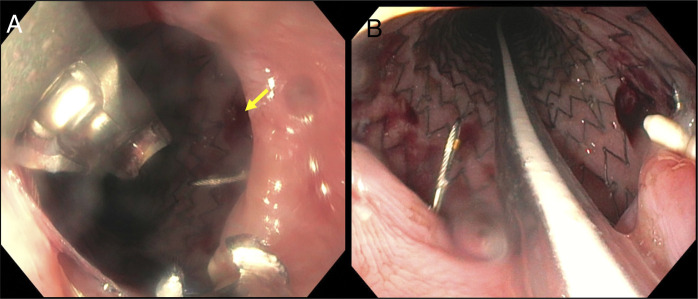
(A) Mucosotomy defect closure by endoscopic suturing (arrow). (B) Fully covered self-expanding metal stent placement with sutures in place.

We performed an urgent esophagogastroduodenoscopy (EGD), which demonstrated a 6-mm mucosal defect at the mucosectomy site with displaced clips located at 32 cm from the incisors (10 cm proximal to the lower esophageal sphincter). The defect was closed with endoscopic sutures (using the OverStitch Endoscopic Suturing System), and we also placed a fully covered esophageal stent (Merit Endotek ALIMAXX Esophageal Stent; 23 mm in diameter × 150 mm length) (Figure 2).

The patient did well on the second and third days; however, despite these interventions, repeated imaging showed a persistent leak. On the third day, we decided to remove the stent, revealing a patent proximal mucosectomy site, for which endoscopic sutures were applied for the second time (Figure 1), and the patient was kept nil per os with total parenteral nutrition.

On the fifth day, a repeat CT and esophagogram continued to show persistent leakage; however, the patient was doing well clinically, so we postponed the next step of management.

**Figure 3.**
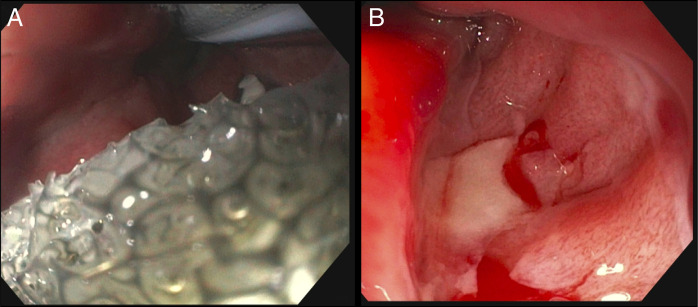
(A) Endoluminal vacuum therapy sponge in situ. (B) The mucosectomy site shows ulcerations with no apparent defect.

Given the ongoing leak, on the eighth postoperative day, we decided to pursue EndoVAC therapy. During EGD, we accessed the mucosotomy site, which revealed a contained leak with significant granulation tissue. After irrigation and suction, an intraluminal EndoVAC sponge was attached to a 16F nasogastric tube and positioned endoscopically at the mucosotomy site under continuous suction at –75 mm Hg (Figure 3A). On the fifth day of EndoVAC, we repeated EGD, and it showed ulceration with no visible defect (Figure 3B), and the esophagogram confirmed complete leak resolution. The patient was discharged on a liquid diet and later advanced to a soft diet with good recovery and complete resolution of dysphagia symptoms on 3 months of follow-up.

## DISCUSSION

Although POEM is generally safe and less invasive than surgical alternatives, complications can occur. Postprocedural mucosal defects leading to esophageal leak are uncommon but represent a serious adverse event that requires timely, multimodal management. Current treatment strategies for mucosal defects include surgical intervention as well as less invasive endoscopic approaches, such as endoscopic stent placement, suturing, clip closure, fibrin glue application, incision of the mucosal flap, and endoscopic internal drainage with pigtail placement.^5,6^

Endoscopic vacuum therapy (EVT, EndoVAC) was first introduced in 2008 and has since been evaluated in multiple studies for the management of gastrointestinal leaks and perforations.^7^ In our case, initial clip closure at the mucosectomy site failed to contain the defect. Subsequent attempts using endoscopic suturing and endoluminal stent placement were also unsuccessful in achieving leak control. EVT was therefore pursued as a salvage therapy and resulted in complete closure of the mucosal defect and resolution of the leak by day 5 post-EVT.

Previous studies have demonstrated the high efficacy of EVT in upper gastrointestinal defects. Binda et al reported success rates ranging from 81.6% to 85%,^5^ while a multicenter cohort study reported an even higher success rate of approximately 89% in the management of esophageal perforations.^8^ Despite its high success rate, EVT is rarely used for post-POEM defects. This is largely due to the widespread availability and reliability of first-line modalities, such as self-expandable metal stents and endoscopic suturing, which are effective in most cases. However, in the small subset of patients who fail these interventions, alternative therapeutic approaches are necessary. In such scenarios, endoscopic vacuum-assisted closure may be particularly advantageous, especially when the defect is associated with a contained cavity, a situation in which self-expandable metal stents may be less effective. Nevertheless, endoscopic vacuum-assisted closure has several limitations, including the need for multiple sequential endoscopic procedures, prolonged hospitalization, nil per os status, and patient discomfort related to the trans-nasal device.^5–8^

While the EVT was pursued earlier in other studies, in our case, EVT was initiated 8 days after defect recognition, following failure of conventional endoscopic therapies, and was maintained for a total duration of 5 days without sponge exchange. While sponge exchange is necessary to prevent tissue growth into the sponge, by the first follow-up the defect had completely closed off; therefore, the sponge exchange was not needed. This treatment duration is notably shorter than the mean duration of 12–30 days reported in previous studies.^7,8^

Previous studies demonstrate considerable variability in the negative pressure settings used for EVT in the management of esophageal leaks, with reported pressures ranging from −20 mm Hg to −125 mm Hg or higher.^9^ These differences are often influenced by factors such as leak location, cavity size, presence of infection, and institutional protocols. We have had good experience with a suction pressure of 75 mm Hg for the management of esophageal leak.

Although EVT has been used in multiple settings before, including post-Z-POEM nonhealing cavities with fistulous track,^10^ to the best of our knowledge, we believe our case is among the first reported uses of EVT for the treatment of a contained esophageal leak following esophageal POEM procedure.

In conclusion, EVT represents a highly effective and minimally invasive therapeutic option for refractory esophageal leaks, with the potential to promote rapid healing and facilitate early clinical recovery.

## DISCLOSURES

Author contributions: M. Elshennawy: collected and analyzed clinical data, conducted the literature review, drafted the original manuscript, and implemented revisions based on coauthor feedback. S. Mahmood: supervised patient management, provided critical intellectual content and substantive revisions to the manuscript, gave final approval of the version to be published, and agrees to be accountable for all aspects of the work in ensuring that questions related to the accuracy or integrity of any part of the work are appropriately investigated and resolved. S. Mahmood is the article guarantor.

Financial disclosure: None to report.

Informed consent was obtained for this case report.

## ABBREVIATIONS:

CT: Computed Tomography
EGD: esophagogastroduodenoscopy
EID: endoscopic internal drainage with pigtail placement
EndoVAC: endoluminal vacuum therapy
EVT: endoscopic vacuum therapy
NG: Nasogastric
POEM: peroral endoscopic myotomy
Z-POEM: Zenker's Peroral Endoscopic Myotomy

## References

[1] Pu XX Huang S Zhong CY . Safety and efficacy of peroral endoscopic myotomy for treating achalasia in pediatric and geriatric patients: A meta-analysis. World J Gastrointest Endosc. 2024;16(10):566–80. 39473543 10.4253/wjge.v16.i10.566 PMC11514429

[2] Stavropoulos SN Iqbal S Modayil R Dejesus D. Per oral endoscopic myotomy, equipment and technique: A step-by-step explanation. Video J Encyclopedia GI Endosc. 2013;1(1):96–100.

[3] Haito-Chavez Y Inoue H Beard KW . Comprehensive analysis of adverse events associated with per oral endoscopic myotomy in 1826 patients: An international multicenter study. Am J Gastroenterol. 2017;112(8):1267–76. 28534521 10.1038/ajg.2017.139

[4] Abdulsada M Sealock RJ Cornwell L Ketwaroo GA. Endoluminal vacuum therapy of esophageal perforations. VideoGIE. 2020;5(1):8–10. 31922071 10.1016/j.vgie.2019.10.004 PMC6945136

[5] Binda C Jung CFM Fabbri S . Endoscopic management of postoperative esophageal and upper GI defects—a narrative review. Medicina (Kaunas, Lithuania). 2023;59(1):136. 36676760 10.3390/medicina59010136 PMC9864982

[6] Cocomazzi F Gentile M Carparelli S Varano L Perri F. Mucosal leak after peroral endoscopic myotomy: What to do? Endoscopy. 2024;56(Suppl 01):E955–6. 39515768 10.1055/a-2446-6638 PMC11548998

[7] Bludau M Fuchs HF Herbold T . Results of endoscopic vacuum-assisted closure device for treatment of upper GI leaks. Surg Endosc. 2018;32(4):1906–14. 29218673 10.1007/s00464-017-5883-4

[8] American Society for Gastrointestinal Endoscopy Technology Committee; Han S Girotra M Abdi M . Endoscopic vacuum therapy. iGIE. 2024;3(3):333–41. 41646140 10.1016/j.igie.2024.06.003 PMC12850756

[9] Jung CFM Müller-Dornieden A Gaedcke J . Impact of endoscopic vacuum therapy with low negative pressure for esophageal perforations and postoperative anastomotic esophageal leaks. Digestion. 2021;102(3):469–79. 32045916 10.1159/000506101

[10] Alsaad AA Massoud D Shrigiriwar A Fayyaz F Mehta A Khashab MA. Endoscopic vacuum therapy for nonhealing cavity with fistulous tract after peroral endoscopic myotomy for Zenker's diverticulum. VideoGIE. 2024;9(3):117–8. 38482472 10.1016/j.vgie.2023.10.017 PMC10928134

